# Cardiac Fibroblast-Specific Knockout of PGC-1α Accelerates AngII-Induced Cardiac Remodeling

**DOI:** 10.3389/fcvm.2021.664626

**Published:** 2021-06-16

**Authors:** Hong-jin Chen, Xiao-xi Pan, Li-li-qiang Ding, Cheng-chao Ruan, Ping-jin Gao

**Affiliations:** ^1^Department of Cardiovascular Medicine, State Key Laboratory of Medical Genomics, Shanghai Key Laboratory of Hypertension, Department of Hypertension, Shanghai Institute of Hypertension, Ruijin Hospital, Shanghai Jiao Tong University School of Medicine, Shanghai, China; ^2^Shanghai Key Laboratory of Bioactive Small Molecules, Department of Physiology and Pathophysiology, School of Basic Medical Sciences, Fudan University, Shanghai, China

**Keywords:** cardiac fibroblast, fibrosis, PGC-1α, cardiac remodeling, AngII

## Abstract

Cardiac remodeling consisted of ventricular hypertrophy and interstitial fibrosis is the pathological process of many heart diseases. Fibroblasts as one of the major cells in the myocardium regulate the balance of the generation and degeneration of collagen, and these cells transform toward myofibroblasts in pathological state, contributing to the remodeling of the heart. Peroxisome proliferator-activated receptor-γ (PPAR-γ) coactivator-1α (PGC-1α) is vital to the function of mitochondria, which contributes to the energy production and reactive oxidative species (ROS)-scavenging activity in the heart. In this study, we found that fibroblast-specific PGC-1α KO induced cardiac remodeling especially fibrosis, and Angiotensin II (AngII) aggravated cardiac fibrosis, accompanied with a high level of oxidative stress response and inflammation.

## Introduction

Cardiac remodeling is the main pathological mechanism of heart failure which is characterized by left ventricular hypertrophy and interstitial fibrosis (1, 2). Hypertension is the most common cause of cardiac remodeling, in which the renin–angiotensin system (RAS) plays an important role. AngII is a key trigger of heart remodeling, which stimulates the expression of TGF-β through the angiotensin type 1 receptor, contributing to cardiac hypertrophy and fibrosis (3). The myocardium is composed of several cell types and vascular and neuronal networks. The types of cells include cardiomyocytes, cardiac fibroblasts (CFs), and endothelial cells. Also, there is abundant and complex extracellular matrix (ECM) in the interstitium, including several types of collagen proteins. Among these, type I collagen proteins account for more than 70% (4), which is critical to maintaining the structural integrity of the heart and generates a stress-tolerant network. CFs are a key source of ECM and are responsible for the homeostasis of the ECM, contributing to tissue repair and fibrosis (5). Acute stimulation or progressive damage induces necrosis or apoptosis of cardiomyocytes, followed by phagocytosis of neighboring cells. Myofibroblasts surround the injury area and produce interstitial collagen, resulting in cardiac remodeling (6, 7), but extensive ECM accumulation will lead to the dysfunction of the myocardium and finally result in heart failure. In fact, there are no activated myofibroblasts in the healthy myocardium, and CFs transfer to myofibroblasts and migrate to the injured area upon cardiac damage (8). In addition, myofibroblasts are also derived from epithelial and endothelial cells through the process of epithelial–mesenchymal transition (EMT) and endothelial–mesenchymal transition (EndMT), respectively (9, 10). Moreover, myofibroblasts are characterized by the expression of alpha-smooth muscle actin (α-SMA), Cofilin, Palladin 4lg, and other markers (11–13). As mentioned above, in physiological conditions, the synthesis and degeneration of collagen are in dynamic equilibrium. CF has been reported to secrete the collagenase and stromelysin, which have the ability to degrade surrounding interstitial collagens (14). In an injured heart, increased synthesis of collagen that exceeds the rate of degradation can lead to cardiac fibrosis. CFs are crucial in regulating the process of cardiac remodeling, but the exact mechanism remains unclear.

Peroxisome proliferator-activated receptor gamma (PPARγ) coactivator-1 alpha (PGC-1α) is an important coactivator of several nuclear receptors and regulates mitochondrial function in various organs and tissues, including the heart, brain, and liver (15). Initially, PGC-1α was found in the brown adipose tissue and skeletal muscle of the mice exposed to cold conditions, and it plays a key role in thermogenesis (16). As the research of PGC-1α moves along, researchers found that activated PGC-1α stimulates mitochondrial oxidative metabolism. Thus, PGC-1α is highly expressed in the heart, brain, and kidney (17–19). The decreased expression of PGC-1α has been found in many diseases, which is often accompanied by a change of metabolic substrate from fatty acid to glucose (20, 21). Previous study has reported that the reduction of PGC-1α exacerbated non-alcohol fatty liver disease which eventually evolved into liver fibrosis. Moreover, this pathological process was accompanied by increased inflammation and oxidative damage (22). In terms of the heart, PGC-1α knockout mice exhibit lower treadmill running times and cardiac function decline after exercise compared to wild-type (WT) mice (23, 24). The synthesis and degeneration of collagen are energy-consuming and enzyme-dependent processes. The myocardium is mainly composed of cardiomyocytes and CFs, and both of them express PGC-1α. Although whole-body PGC-1α knockout mice have been reported to have heart dysfunction, the role of CF-specific PGC-1α in the heart is still unclear.

In the present study, we investigated the role of PGC-1α in CF. The activated fibroblasts have been considered the primary cause of cardiac fibrosis, and PGC-1α is essential to maintaining the function of CFs. Thus, we suppose that PGC-1α in CFs takes part in regulating the process of heart remodeling. Herein, we utilized the Cre-Loxp system to construct CF-specific PGC-1α knockout mouse and further examined its effects on the heart. In addition, we cultured CFs *in vitro* to study the function of PGC-1α.

## Materials and Methods

### Animal Study

PGC-1α^flox^ (C57BL/6J background), SM22α^Cre/ER^ (C57BL/6J background) mice were obtained from the Jackson Laboratory (Bar Harbor, ME, USA). PGC-1α^flox/flox^ mice were bred with SM22α^Cre/ER^ mice to produce SM22α^Cre/ER^;PGC-1α^flox/−^ heterozygote mice. Then, heterozygote mice were bred with PGC-1α^flox/flox^ mice to produce SM22α^Cre/ER^;PGC-1α^flox/flox^ (SP) homozygote mice. The WT mice and SP mice we used in the study are all 2- to 3-month-old male mice. The animal study was approved in accordance with institutional guidelines established by the Committee of Ethics on Animal Experiments at Shanghai Jiao Tong University School of Medicine.

### Identification of Transgenic Mice

SP mice were identified by tail DNA PCR using primers for SM22α and PGC-1α (Supplementary Table 2). Mouse tail DNA was extracted by Mouse Tail Genomic DNA Kit (CWBIO). PCR was performed for 34 cycles with each cycle at 95°C for 30 s, 62°C for 30 s, and 72°C for 30 s. Finally, DNA agarose gel electrophoresis was used to identify mouse genotype (Supplementary Figure 1).

### AngII-Induced Cardiac Remodeling

WT mice and SP mice were assigned to sham group and AngII group, respectively. AngII group mice were implanted subcutaneously with osmotic mini-pumps (Alzet, model: 2004, ALZET® Osmotic Pumps, Cupertino, CA, USA) to deliver AngII (1.44 mg/kg/day) for 28 days. The sham group received the same amount of saline.

### Cell Culture

Primary CFs were isolated from 3 to 4-day-old male mice, as described previously (25). Cells were cultured in DMEM with 20% FBS and 1% penicillin and streptomycin. Then, CFs in the AngII group were stimulated by AngII (10^−7^ M) for 12 h.

### Recombinant Lentiviruses

Lentiviruses carrying small hairpin RNA (shRNA) were produced and purified by GeneChem. The PGC-1α shRNA sequence was ACTATTGAGCGAACCTTAA, and the no-target control shRNA sequence was TTCTGCGAACGTGTCACGT. The viruses were used to infect CFs for 72 h (MOI = 10).

### Histology and Immunostaining

Aortas fixed in formalin and embedded in paraffin were sectioned at 5 μm. Masson or wheat germ agglutinin (WGA) staining was performed using standard procedures. For immunofluorescence staining, the paraffin-embedded and frozen sections of the heart were incubated with primary antibodies for α-SMA (1:100) (19245S, CST, Danvers, MA, USA), Col1a1 (1:100) (GB11022, Servicebio, Wuhan, China), and F4/80 (1:100) (ab6640, Abcam, Cambridge, UK). Antigen retrieval of the paraffin section was obtained by heating the tissue slides in 0.01 M citrate buffer, pH 6.0, at 100°C for 5 min.

### Quantitative Real-Time PCR

Total RNA was extracted from tissues and cultured cells using TRIzol (Invitrogen, Carlsbad, CA, USA) followed by chloroform extraction according to the manufacturer's protocol. Total RNA was reverse transcribed into single-stranded cDNA by incubation with reverse transcriptase (EZBioscience, Roseville, MN, USA). Real-time qRT-PCR was performed with SYBR Premix Ex Taq kits with ROX (TaKaRa) according to the manufacturer's instructions. Signals were detected on an ABI PRISM 7900 machine (Applied Biosystems, Foster City, CA, USA). β-Actin was used as a standard reference. Reactions were done at 95°C for 30 s followed by 40 cycles of 95°C for 5 s and 60°C for 30 s. Sequences of primers used in this study are provided in Supplementary Table 1.

### Western Blotting

Frozen tissues were powdered and then homogenized in ice-cold RIPA buffer (50 mM Tris–HCl (pH 7.4); 10% Nonidet P-40; 0.25% sodium deoxycholate; 150 mM NaCl; 1 mM EDTA; 0.5 M NaF; 10 mM sodium pyrophosphate) supplemented with Protease Inhibitor Cocktail (BioTool Swiss, Kirchberg, Switzerland) and a phosphatase inhibitor (BioTool). Cultured cells were directly lysed in RIPA buffer. Proteins were applied into 10% SDS-PAGE gel and blotted onto a PVDF membrane (Merck Millipore, Burlington, MA, USA). Furthermore, blots were incubated with α-SMA (1:1,000) (19245S, CST), TGF-β (1:1,000) (3711S, CST), Col1a1 (1:1,000) (GB11022, Servicebio), iNOS (1:1,000) (ab178945, Abcam), and GAPDH (1:1,000) (5174S, CST) antibodies overnight at 4°C and then incubated with an HRP-conjugated antibody for 2 h at room temperature. The signal was detected by chemiluminescence.

### Statistics

Differences between two independent groups were determined using Student's *t*-test (two-tailed). To compare more than two groups, one-way analysis of variance (ANOVA) was conducted followed by *post-hoc* Dunnett's testing for multiple-group comparison by GraphPad Prism (GraphPad Software, San Diego, CA, USA). Data represent mean ± SEM. The significant level was set at p < 0.05.

## Results

### CF-Specific PGC-1αKO Aggravates Cardiac Fibrosis

**Figure 1:**
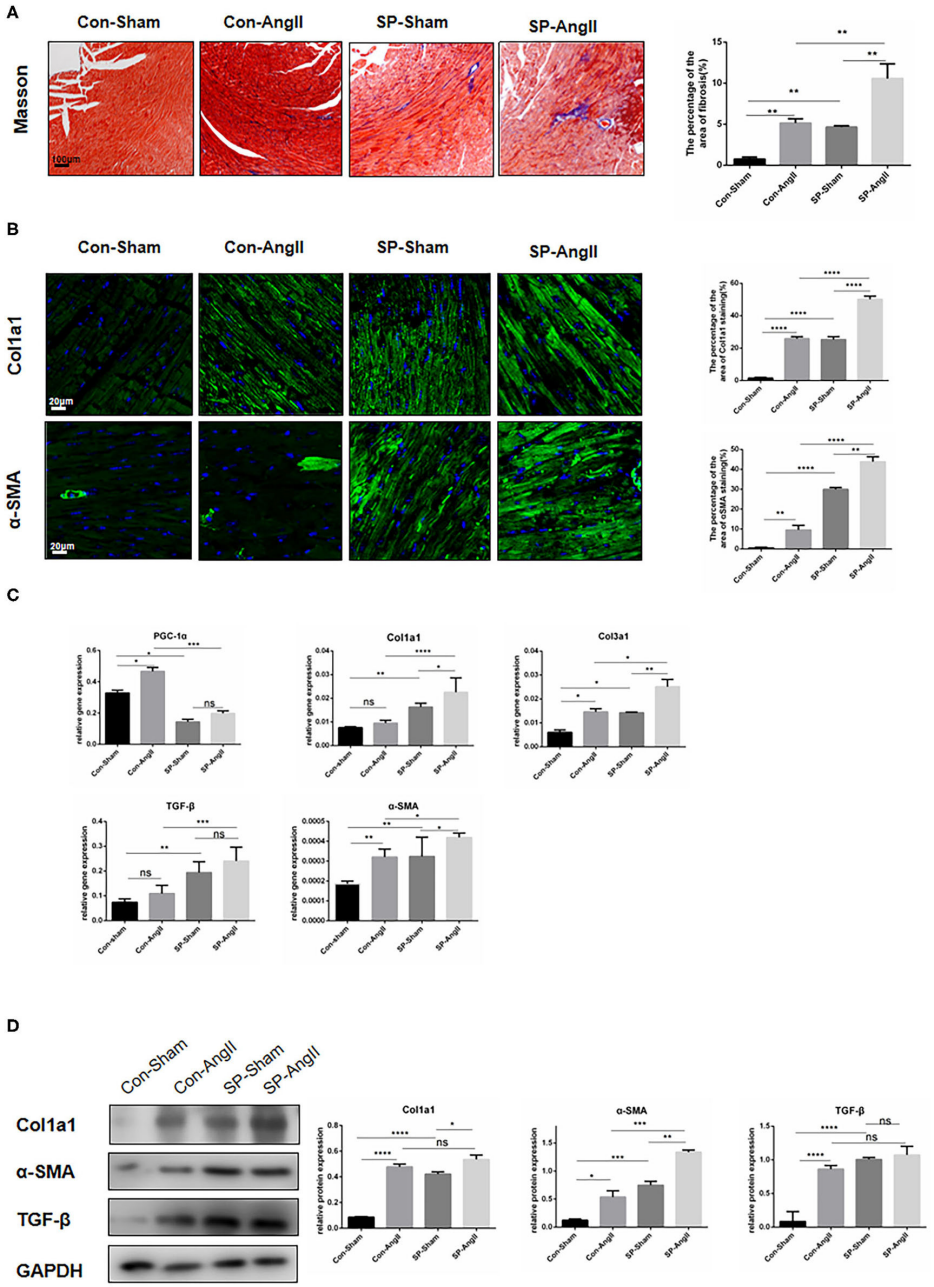
Cardiac fibroblast-specific PGC-1αKO aggravates cardiac remodeling. **(A)** Representative cross sections of the heart stained for Masson and quantitative analysis of the area of fibrosis (Masson staining). **(B)** Representative immunofluorescent staining of Col1a1 and α-SMA and quantitative analysis of immunofluorescent staining of Col1a1 and α-SMA. **(C)** qPCR analysis of mRNA expression levels of *PGC-1*α and fibrotic genes (*Col1a1, Col3a1*, α*-SMA, TGF-*β). **(D)** Representative Western blot and analysis of the expression of fibrotic proteins (Col1a1, α-SMA, TGF-β). N.S. indicates no significant difference. *p < 0.05, **p < 0.01, ***p < 0.001, ****p < 0.0001. Data represent mean ± SEM (*n* = 5 per group).

To determine whether the absence of PGC-1α in CFs could impact on heart health, WT and CF-specific PGC-1α KO mice were treated with AngII for 28 days. SM22α^Cre/ERT^;PGC-1α^flox/flox^ (SP) mice were utilized to study the role of CF-specific PGC-1α KO in cardiac remodeling, including interstitial fibrosis and cardiac hypertrophy. Masson's trichrome (Masson) staining was performed to analyze interstitial fibrosis. Histology analysis showed that WT mice developed significant heart fibrosis under administration of AngII for 28 days. More importantly, dramatic cardiac fibrosis was observed in both SP mice with and without the stimulation of AngII, and the SP-AngII group exhibited a higher level of fibrosis. Furthermore, SP mice developed more severe fibrosis than WT mice after AngII infusion (Figure 1A). In immunofluorescent staining of Col1a1 and α-SMA, increased expressions of Col1a1 and α-SMA were detected in WT mice after the stimulation of AngII. CF-specific PGC-1α KO induced collagen deposition in the heart, and AngII infusion aggravated it (Figure 1B). Then, qPCR was performed to explore the level of gene expression. The mRNA levels of fibrosis markers including collagen type I alpha 1 chain (*Col1a1*), collagen type III alpha 1 chain (*Col3a1*), *TGF-*β, and α*-SMA* were increased in specific PGC-1αKO mice compared to WT mice. Under the stimulation of AngII, the heart of SP-AngII mice presented upregulation of fibrosis-associated genes compared with that of control-AngII mice (Figure 1C). PGC-1αKO promoted the expressions of Col1a1, TGF-β, and α-SMA in heart (Figure 1D). These results indicated that PGC-1α plays an important role in maintaining the normal function of CFs, which can further influence heart health.

### CF-Specific PGC-1αKO Aggravates Cardiac Hypertrophy

**Figure 2:**
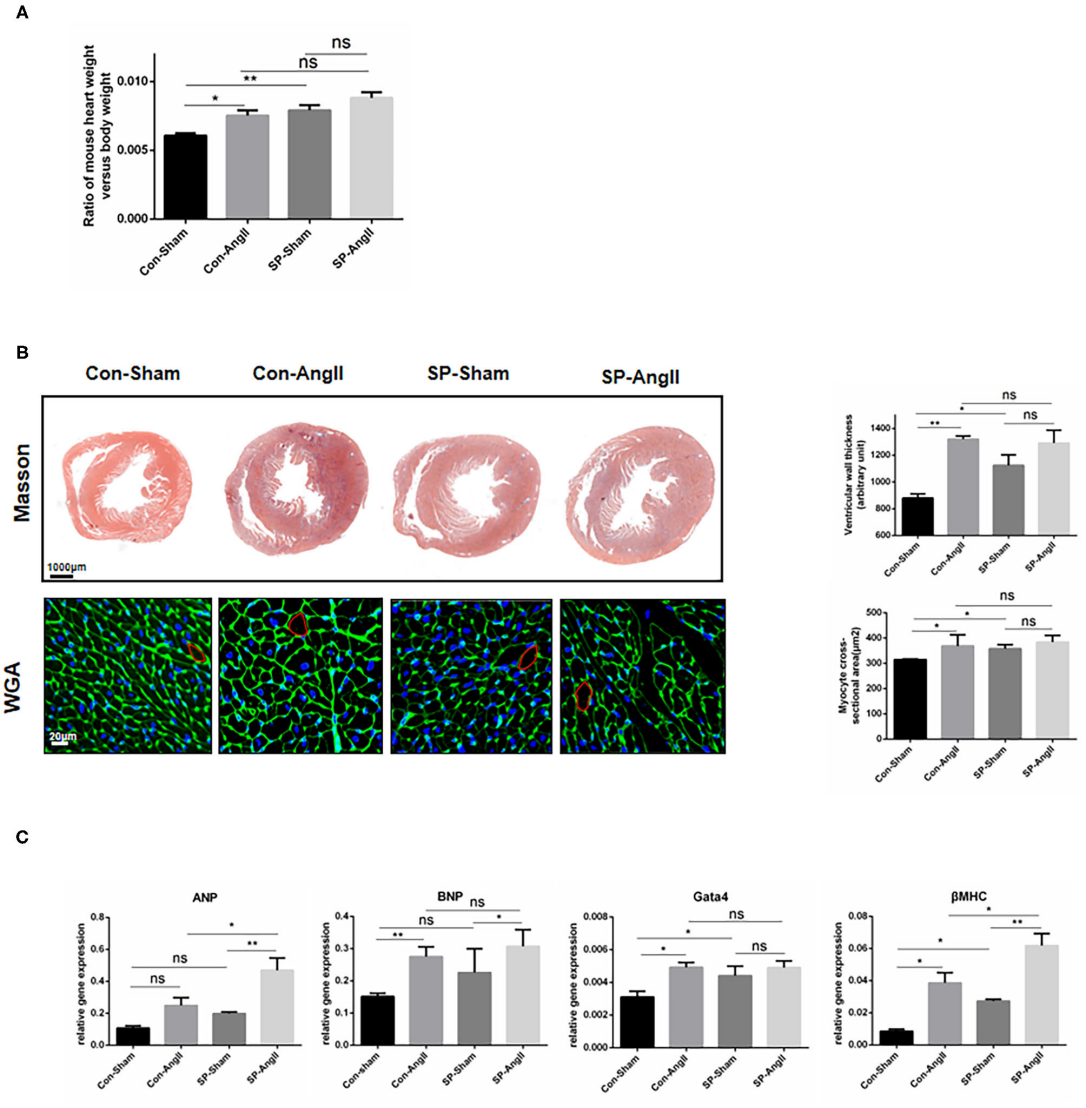
Cardiac fibroblast-specific PGC-1αKO aggravates cardiac hypertrophy. **(A)** Quantitative analysis of the ratio of the weight of mouse heart to body weight. **(B)** Representative cross sections of the heart stained for Masson and WGA. Quantitative analysis of the thickness of ventricular wall (Masson staining) and cardiomyocyte size (WGA staining). **(C)** qPCR analysis of mRNA expression levels of hypertrophic markers (ANP, BNP, Gata4, βMHC). N.S. indicates no significant difference. *p < 0.05, **p < 0.01. Data represent mean ± SEM (*n* = 5 per group).

To study the possible influence of fibroblast-specific knockout of PGC-1α in cardiac hypertrophy, the following experiments were carried out. Both AngII treatment and CF-PGC1a KO induced the increase in the heart weight to body weight ratio, but there was no significant difference between the SP-sham and SP-AngII groups (Figure 2A). Masson and WGA staining results showed that ventricular wall thickness and cardiomyocyte size were increased in both WT mice and SP mice after the stimulation of AngII. Without the infusion of AngII, there were elevated ventricular wall thickness and cardiomyocyte size in SP mice compared with WT mice (Figure 2B). Besides, CF-specific PGC-1α KO resulted in elevated mRNA expression of hypertrophic relative factors, including atrial natriuretic peptide (*ANP*), atrial natriuretic peptide (*ANP*), brain natriuretic peptide (*BNP*), *Gata4*, and myosin heavy chain β (β*MHC*) compared with WT mice (Figure 2C). These findings suggested that CF-specific PGC-1α KO aggravated cardiac hypertrophy, which was independent of the stimulation of AngII.

### CF-Specific PGC-1αKO Activates the Oxidative Stress Response in the Heart

**Figure 3:**
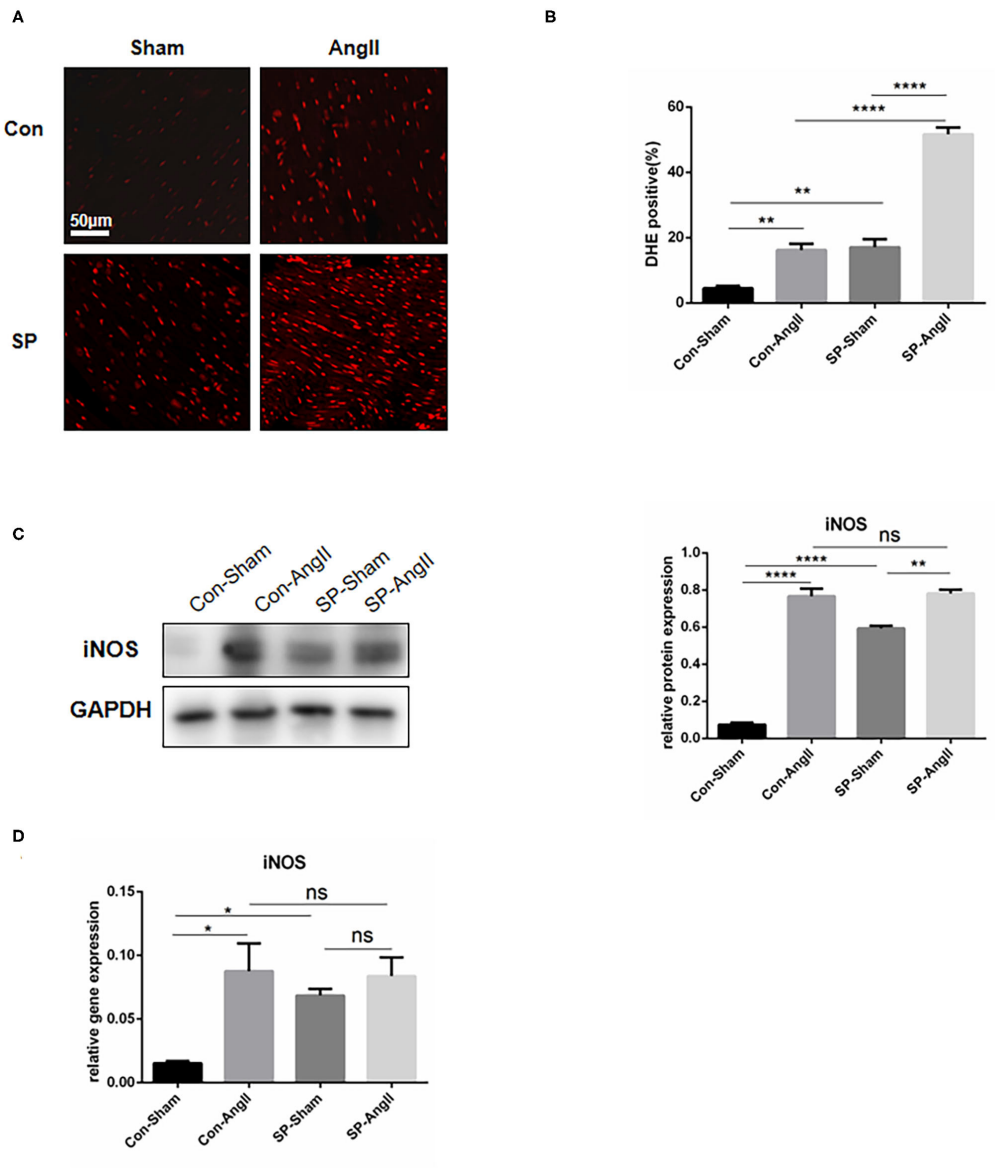
Cardiac fibroblast-specific PGC-1αKO activates the oxidative stress response in the heart. **(A)** Representative cross sections of the heart stained for DHE. **(B)** Quantitative analysis of reactive oxidative stress as assessed by DHE staining. **(C)** Representative Western blot and analysis of the expression of iNOS. **(D)** qPCR analysis of the mRNA expression levels of *iNOS*. N.S. indicates no significant difference. *p < 0.05, **p < 0.01. Data represent mean ± SEM (*n* = 3–5 per group).

PGC-1α is considered as a suppressor in oxidative stress response (26). To explore whether the knockdown of PGC-1α in CFs could aggravate the oxidative stress in heart, the following experiments were carried out. Dihydroethidium (DHE) staining was utilized to examine the level of ROS. The results showed that the intensity and the proportion of DHE-positive cells were elevated in both WT mice and CF-PGC-1αKO mice after the stimulation of AngII. The heart of SP mice exhibited a higher level of ROS production than that of WT mice in both sham and AngII infusion (Figures 3A,B). The protein expression of inducible nitric oxide synthase (iNOS) was increased in both WT mice and SP mice after the stimulation of AngII. Moreover, the content of iNOS was at a higher level in SP mice compared to WT mice without the treatment of AngII (Figure 3C). In addition, the mRNA level of iNOS in the heart was measured. The results of analysis were identical to the expression of protein (Figure 3D). These data showed that PGC-1α knockdown in CFs aggravated the oxidative stress response in the heart.

### CF-Specific PGC-1αKO Aggravates the Inflammatory Response in the Heart

**Figure 4:**
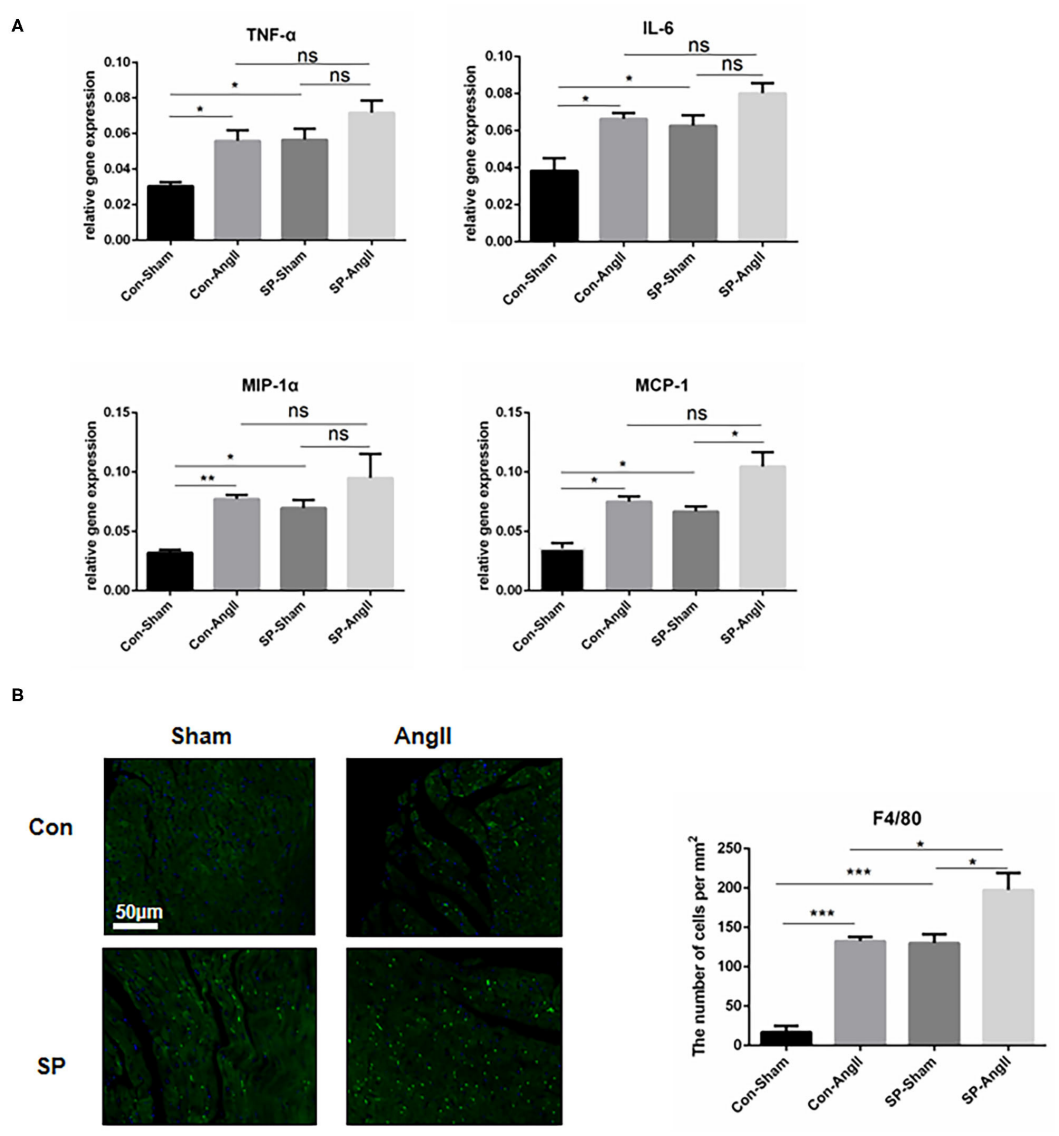
Cardiac fibroblast-specific PGC-1αKO aggravates the inflammatory response in the heart. **(A)** qPCR analysis of the mRNA expression levels of inflammatory genes (*TNF-*α, *IL-6, MCP-1, MIP-1*α). N.S. indicates no significant difference. **(B)** Representative cross sections of the heart stained for F4/80 and quantitative analysis of the number of positive cells. *p < 0.05, **p < 0.01, ***p < 0.001. Data represent mean ± SEM (*n* = 5 per group).

Inflammation is an important process in cardiac remodeling. The accumulation of inflammatory cells in the heart is closely associated with development of cardiac fibrosis. Furthermore, the activation of fibroblasts is responsive for elevated pro-inflammatory cytokines, which promotes the proliferation and migration of myofibroblasts and accelerates the synthesis of collagen (12, 27). To confirm the effect of PGC-1αKO in CFs on inflammation, qPCR was carried out to examine the mRNA levels of genes coding pro-inflammatory cytokines, including tumor necrosis factor-α (*TNF-*α), interleukin 6 (*IL-6*), macrophage inflammatory protein (*MIP-1*α), and monocyte chemotactic protein 1 (*MCP-1*). After the treatment of AngII, the levels of the above genes were elevated dramatically in WT mice. CF-PGC1a KO mice showed upregulation of *TNF-*α, *IL-6, MIP-1*α, and *MCP-1* compared to WT mice, and AngII enhanced the MCP-1 expression in SP-AngII mice (Figure 4A). Consistent with this, there was more amount of macrophage infiltration in the heart of SP mice than WT mice. Moreover, AngII induced inflammation response (Figure 4B). Based on the results, we concluded that PGC-1α in CFs might take part in the regulation of inflammation.

### PGC-1α KO in CF Promotes the Expression of Fibrosis-Related Genes

**Figure 5:**
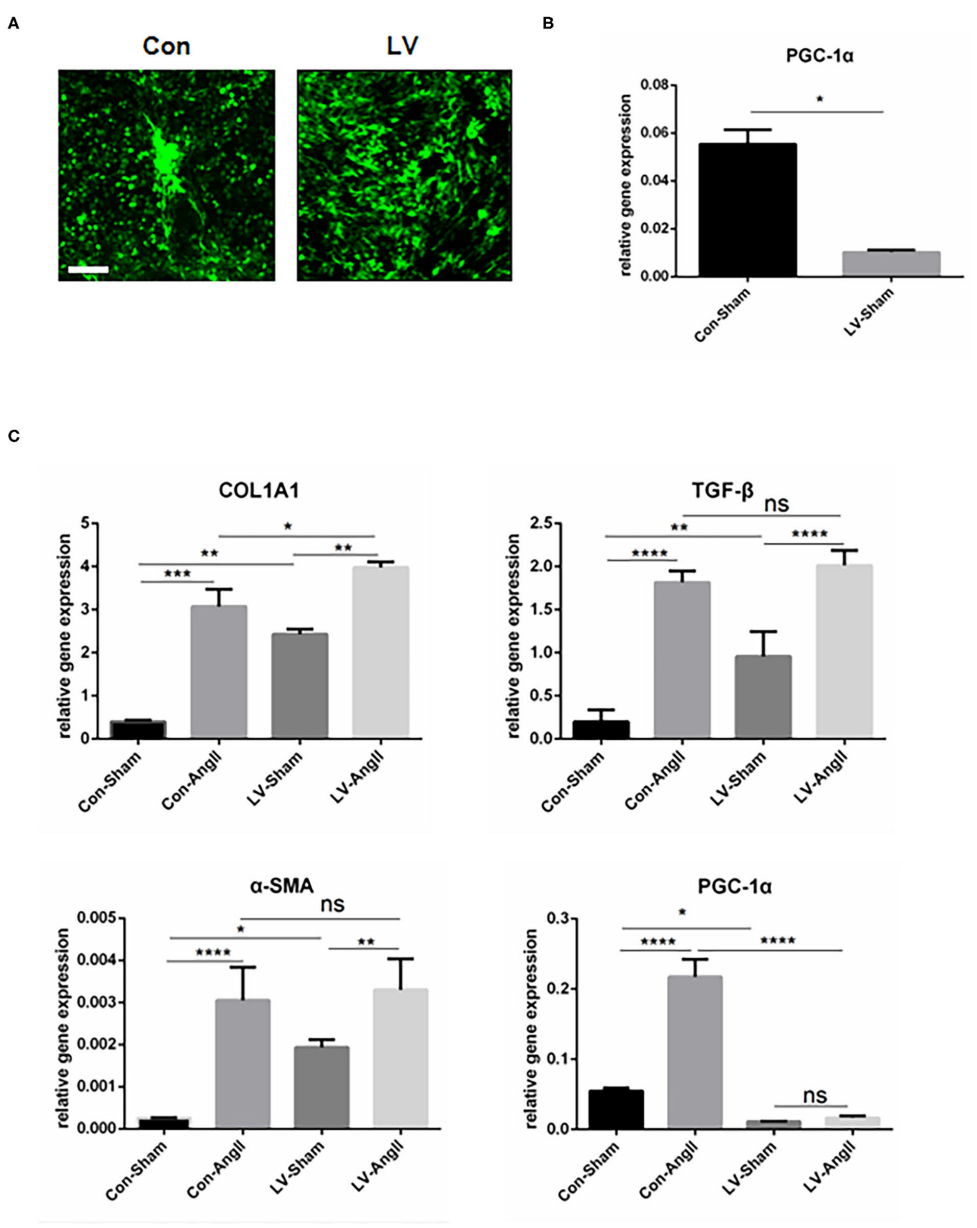
PGC-1α KO in cardiac fibroblast promotes the expression of fibrosis-related genes. **(A)** Representative CF treated with PGC-1α-knockdown lentivirus (LV) and control virus (Con). **(B)** qPCR analysis of the mRNA expression levels of PGC-1α in the LV and Con CF groups. **(C)** qPCR analysis of mRNA expression levels of fibrotic genes (*Col1a1*, α*-SMA, TGF-*β). N.S. indicates no significant difference. *p < 0.05, **p < 0.01, ***p < 0.001. Data represent mean ± SEM (*n* = 3 independent experiments).

To rule out the effect of cardiomyocytes, CFs were cultured *in vitro* to find direct evidence that PGC-1α plays a vital role in maintaining the function of CFs. Lentivirus PGC-1α RNA interference (RNAi) was used to knock down PGC-1α expression in CFs (Figures 5A,B). Without the stimulation, cells knocked down of PGC-1α were detected to have higher mRNA levels of *Col1a1, TGF-*β, and α*-SMA* at baseline. AngII stimulation resulted in an increased expression of fibrosis markers, including *Col1a1, TGF-*β, and α-SMA in both experimental group and control group. Also, there is no significant difference in the expression of TGF-β and α-SMA between Con-AngII and LV-AngII cells (Figure 5C). These results indicated that PGC-1α may be involved in mediating the activation of CFs.

## Discussion

An increasing number of studies have emphasized the relationship between heart remodeling and metabolism. PGC-1α is an important regulator of mitochondrial biology and energy metabolism, and the repression of PGC-1α accelerates cardiac dysfunction and the clinical signs of heart failure (24). Some mechanistic studies have shown that the protective role of PGC-1α in heart requires the activation of Sirtuin 1 (SIRT1), which is a redox-sensitive enzyme. Furthermore, the SIRT1-PGC-1α axis is vital to the performance of cardiac protection through decreasing inflammation, oxidative stress, fibrosis, and so on (28, 29). However, the underlying mechanism of PGC-1α-mediated cardiac remodeling is still unclear. Herein, we unveil the specific role of PGC-1α in regulating the function of CFs, which accelerates cardiac remodeling including cardiac fibrosis and cardiomyocytes hypertrophy.

Reduced PGC-1α is associated with impaired mitochondrial biology in heart diseases and energy expenditure (30). PGC-1α is also considered as a vital regulator of the scavenging of ROS. There is research reporting that ROS accelerates the deposition of collagen through the activation of p53, which results in severe fibrosis (31). In this study, ROS levels were increased in WT mice after AngII infusion, which has been proved to be associated with inhibition of PGC-1α-activated catalase expression (32). Our data showed a slight increase in PGC-1α expression in the heart of WT mice after treatment with AngII. More importantly, AngII failed to enhance PGC-1α expression in the heart of SP mice, which suggested that AngII-induced the PGC-1α upregulation in whole heart tissue which might be attributed to the impact of CF on other cells. Hence, we proposed that the absence of PGC-1α in CFs exerts impact on other cells in the heart, leading to the aggravation of cardiac remodeling. In addition, PGC-1α is an essential component of inflammatory response. Overexpression of PGC-1α inhibits the production of pro-inflammatory cytokines and promotes the secretion of anti-inflammatory cytokines in the heart, preventing the heart from getting damaged (33–35). In our study, CF-specific PGC-1α knockdown promoted the expression of pro-inflammatory cytokines in the heart, which indicated that PGC-1α in CFs took part in the inflammatory response. Previous report has demonstrated that pro-inflammatory cytokines facilitated the transition of CFs to myofibroblasts (8). According to the research, there are only ~2% activated CFs in the adult heart (36). Our data shows that in CF-specific PGC-1α KO mice, plenty of activated CFs convert into myofibroblasts even without the stimulation of AngII by detecting the high expression of fibrosis markers and cardiac hypertrophy markers. All these data show that PGC-1α in CFs, as a versatile factor, plays a vital role in regulating cardiac remodeling. On the contrary, several researches demonstrate that overexpression of PGC-1α beyond physiological content leads to mitochondrial proliferation and myofibrillar displacement, which finally contributes to cardiac failure (30, 37). Thus, maintaining PGC-1α in a physiological stage is crucial for cardiovascular health. Our results exhibited that both AngII and PGC-1α KO have an influence on cardiac remodeling, which may indicate that PGC-1α and AngII perform through an overlapping pathway. The aforesaid SIRT1 is an essential protein in regulating the fibrosis-related pathway in many organs through regulating gene transcription (38, 39). AngII-induced cardiovascular remodeling is reported to be closely related to the reduction of SIRT1. On the other hand, overexpression of SIRT1 suppresses the ROS-induced p38/mitogen-activated protein kinase (MAPK) pathway, which promotes the activation of CFs (40, 41). Moreover, as mentioned before, PGC-1α plays a protective role through cooperating with the activated SIRT1 (29). AngII and PGC-1α might affect the function of SIRT1, leading to the activation of the p38/MAPK pathway and the transition of CFs. There are some limitations in our study. It remains uncertain whether overexpression of PGC-1α could improve cardiac remodeling. Furthermore, the underlying mechanism that CF-specific knockout of PGC-1α participates in cardiac remodeling remains unclear, which still needs further study.

In conclusion, we have discovered the PGC-1α-mediated pathology of cardiac remodeling, especially cardiac fibrosis. We propose that the absence of PGC-1α in CFs impairs the balance of the synthesis and degeneration of collagen through regulating ROS production and inflammation, leading to deposition of collagens and cardiac remodeling. Our findings reveal that PGC-1α is critical for the cardiac fibrosis by multiple fibrogenic pathways.

## Data Availability Statement

The original contributions presented in the study are included in the article/Supplementary Material, further inquiries can be directed to the corresponding author/s.

## Ethics Statement

The animal study was reviewed and approved by the Committee of Ethics on Animal Experiments at Shanghai Jiao Tong University School of Medicine.

## Author Contributions

X-xP and C-cR designed the study. H-jC performed the experiments, analyzed the data, and wrote the manuscript. L-l-qD conducted the *in vitro* experiments. All authors contributed to the article and approved the submitted version.

## Conflict of Interest

The authors declare that the research was conducted in the absence of any commercial or financial relationships that could be construed as a potential conflict of interest.
