# Supplementary material for: Cardiac Fibroblast-Specific Knockout of PGC-1α Accelerates AngII-Induced Cardiac Remodeling
Source: Front Cardiovasc Med. 2021 Jun 16;8:664626. doi: 10.3389/fcvm.2021.664626 (PMC8242582; doi:10.3389/fcvm.2021.664626)
Supplement: Supplementary file 1 [file Data_Sheet_1.docx]

Supplementary Material

# Supplementary Figures and Tables

Table. S1 qPCR primer sequences.

| Gene | Forward(5’-3’) | Reverse (5’-3’) |
| --- | --- | --- |
| Col1a1 | GAGCGGAGAGTACTGGATCG | TACTCGAACGGGAATCCATC |
| TGFβ | CAACAATTCCTGGCGTTACCTTGG | GAAAGCCCTGTATTCCGTCTCCTT |
| αSMA | CTGACAGAGGCACCATGAA | AGAGGCATAGAGGGACAGCA |
| ANP | TCTTCCTCGTCTTGGCCTTT | CCAGGTGGTCTAGCAGGTTC |
| BNP | TGGGAGGTCACTCCTATCCT | GGCCATTTCCTCCGACTTT |
| βMHC | CGGACCTTGGAAGACCAGAT | GACAGCTCCCCATTCTCTG |
| PGC1α | GAAGTGGTGTAGCGACCAATC | AATGAGGGCAATCCGTCTTCA |
| Gata4 | CCCTACCCAGCCTACATGG | ACATATCGAGATTGGGGTGTCT |
| Col 3a1 | CTGTAACATGGAAACTGGGGAAA | CCATAGCTGAACTGAAAACCACC |
| MIP-1α | TTCTCTGTACCATGACACTCTGC | CGTGGAATCTTCCGGCTGTAG |
| MCP-1 | TTCTCTGTACCATGACACTCTGC | CGTGGAATCTTCCGGCTGTAG |
| TNF-α | CCCTCACACTCAGATCATCTTCT | GCTACGACGTGGGCTACAG |
| IL-6 | TAGTCCTTCCTACCCCAATTTCC | TTGGTCCTTAGCCACTCCTTC |
| β -actin | CTAAGGCCAACCGTGAAAAGAT | GGGACAGCACAGCCTGGAT |
| iNOS | GAACTGTAGCACAGCACAGGAAAT | CGTACCGGATGAGCTGTGAAT |

Table. S2 PCR primer sequences.

| Gene | Forward(5’-3’) | Reverse (5’-3’) |
| --- | --- | --- |
| PGC1α | TCCAGTAGGCAGAGATTTATGAC | TGTCTGGTTTGACAATCTGCTAGGTC |
| SM22α | CTCAGAGTGGAAGGCCTGCTT | GGCGATCCCTGAACATGTCC |

Figure. S1 The results of DNA agarose gel electrophoresis

**(A)**The gel electrophoresis of PCR amplification of SM22α (220bp). Lane 1 and Lane 3 are positive. Lane 2 is negative. **(B)**The gel electrophoresis of PCR amplification of PGC-1α(400bp). Lane 1 and Lane 3 are heterozygote (PGC-1α^flox/-^); Lane 2 is homozygote(PGC-1α^flox/flox^). bp: base pair; M:marker of DNA ladder.

**
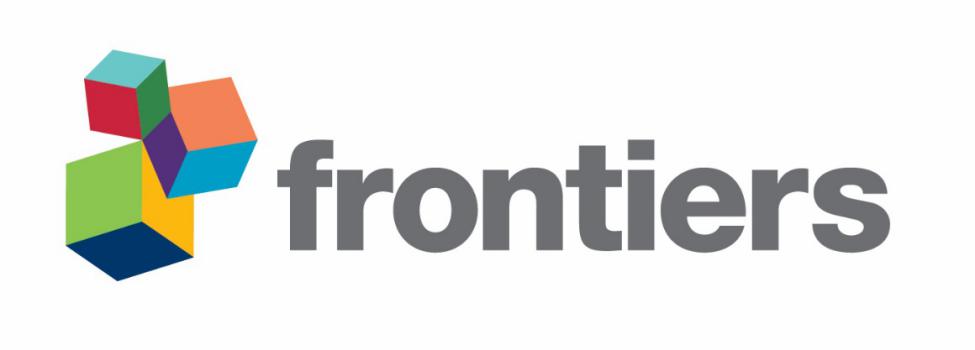
**
