# Supplementary figures and images for: Cardiac Fibroblast-Specific Knockout of PGC-1α Accelerates AngII-Induced Cardiac Remodeling
Source: Front Cardiovasc Med. 2021 Jun 16;8:664626. doi: 10.3389/fcvm.2021.664626 (PMC8242582; doi:10.3389/fcvm.2021.664626)

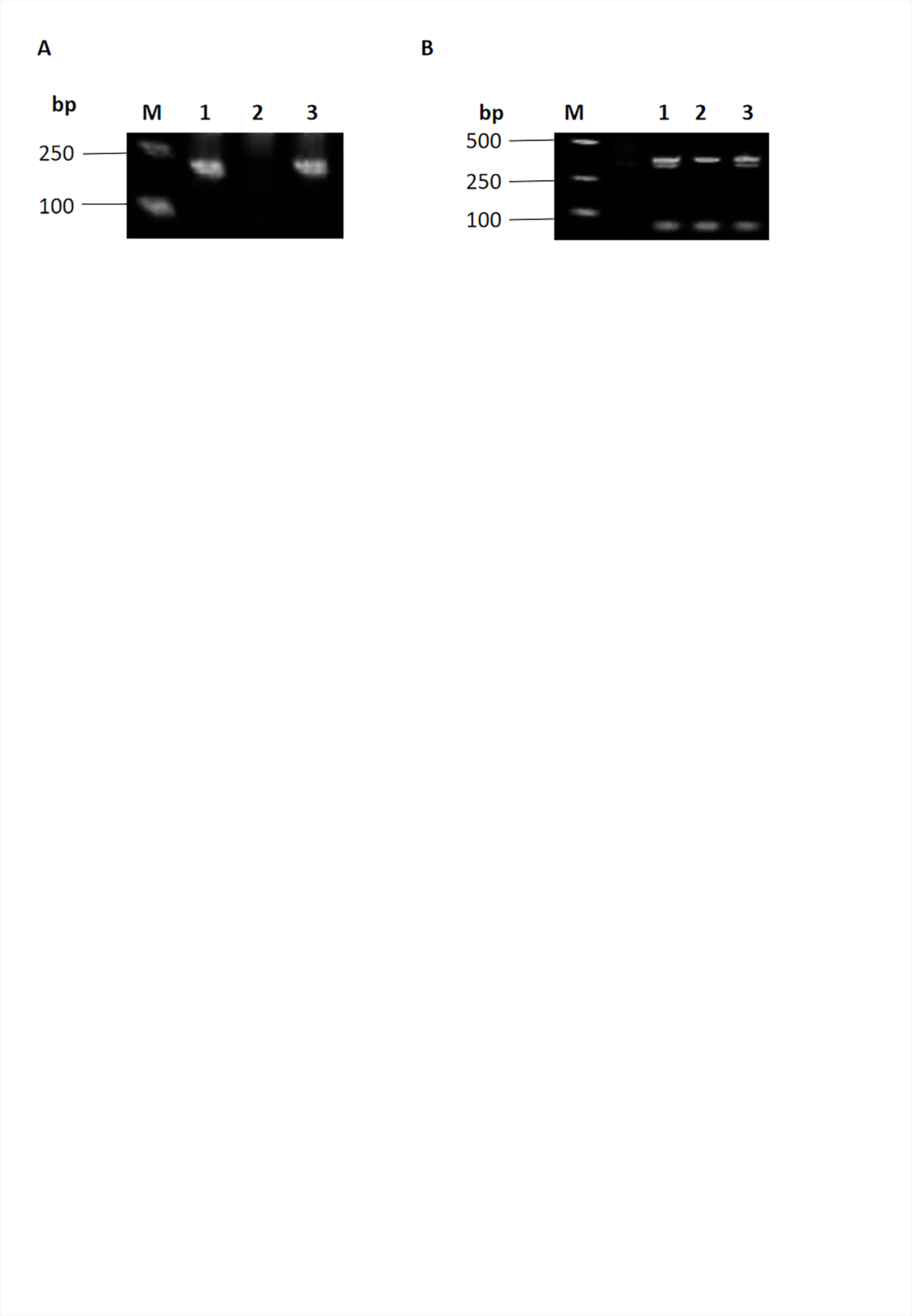

Supplement: Supplementary file 2 [file Image_1.tif]

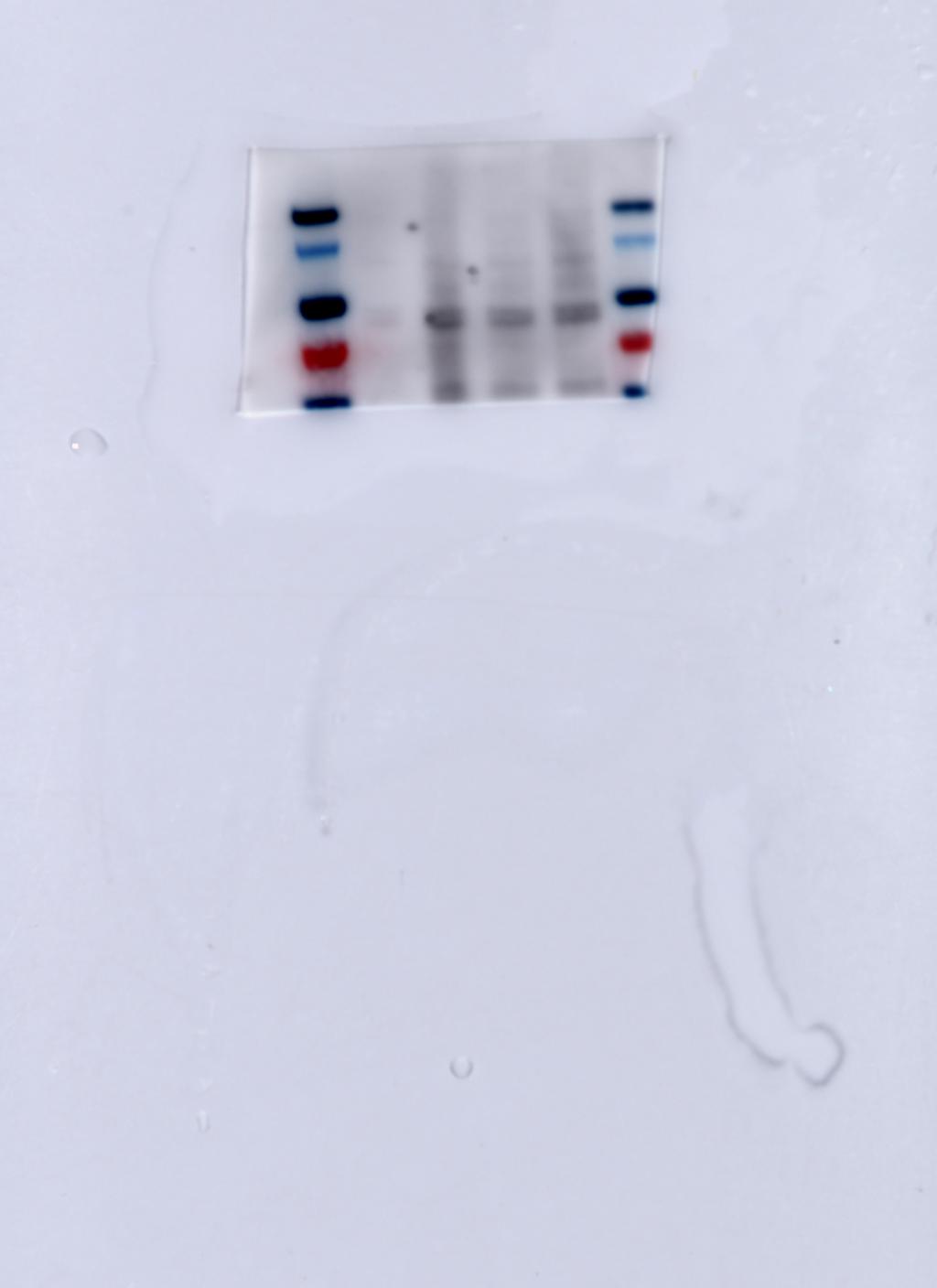

Supplement: Supplementary file 3 [file Image_2.jpeg]

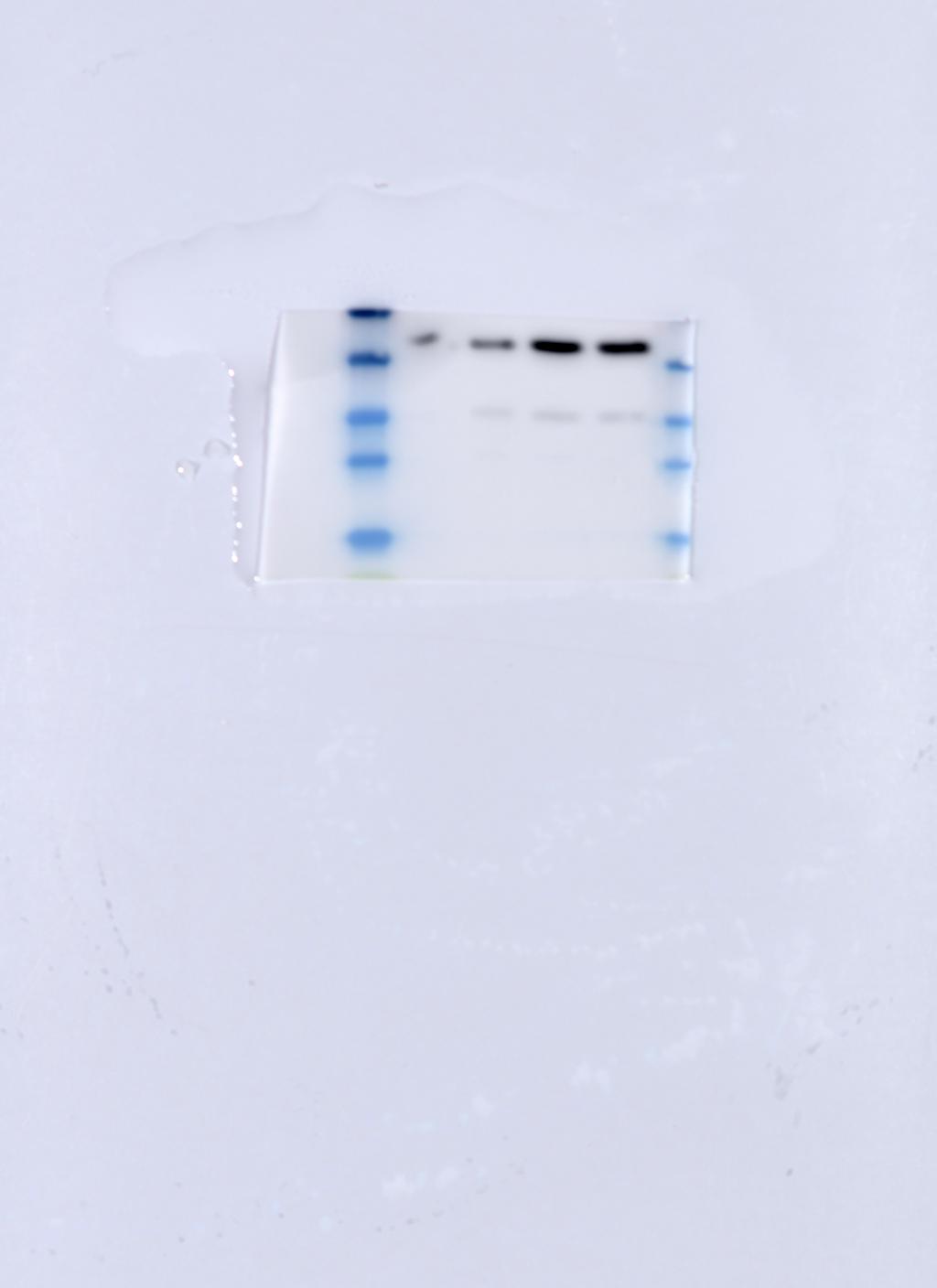

Supplement: Supplementary file 4 [file Image_3.jpeg]
